# Hantaviral therapeutics: life cycle targets and chemotherapeutic interventions

**DOI:** 10.3389/fcimb.2026.1972830

**Published:** 2026-10-16

**Authors:** Deren Zehra Kocabiyik, Jansen de Araujo, Edison Luiz Durigon, Carsten Wrenger

**Affiliations:** 1 Unit for Drug Discovery, Department of Parasitology, Institute of Biomedical Sciences, University of São Paulo, São Paulo, Brazil; 2 Institut Pasteur de São Paulo, São Paulo, Brazil; 3 Department of Microbiology, Institute of Biomedical Sciences, University of São Paulo, São Paulo, Brazil

**Keywords:** antiviral discovery, drug development, orthohantavirus, chemotherapeutics, phytochemicals, viral entry inhibitors, RNA-dependent RNA polymerase (RdRp) inhibitors, viral life cycle targets

## Abstract

Orthohantaviruses represent a persistent global health threat, being responsible for life-threatening diseases such as Hemorrhagic Fever with Renal Syndrome (HFRS) and Hantavirus Cardiopulmonary Syndrome (HCPS). Despite high case fatality rates and recent outbreaks, there are currently no universally licensed antivirals or vaccines. Clinical management remains heavily reliant on supportive care and repurposed drugs, which often exhibit limited efficacy and dose-limiting toxicities. This review examines rational drug design strategies targeting critical steps in the viral life cycle, specifically Gn/Gc-mediated cellular entry and RNA-dependent RNA polymerase (RdRp)-driven genome replication. We evaluate the current landscape of viral inhibitors, including peptidomimetics, nucleoside analogues, and naturally derived compounds. To accelerate the discovery of novel treatments, we advocate for the integration of modern *in silico* screening to evaluate extensive chemical and natural product libraries. These broad compound collections offer a structurally diverse and largely untapped reservoir for antiviral development. Ultimately, expanding preclinical testing to include globally neglected variants will be essential to develop broad-spectrum hantaviral therapeutics capable of mitigating future epidemic threats.

## Introduction

The April 2026 Andes hantavirus outbreak on a cruise ship served as a reminder of the ongoing challenges pathogens pose to global public health (Andes Virus Outbreak Working Group, 2026; Centers for Disease Control and Prevention, 2026; World Health Organization, 2026b). The high case fatality rate highlights that there are currently no universally licensed antivirals or vaccines against orthohantavirus infections (World Health Organization, 2026a). These rodent-borne pathogens belong to the family *Hantaviridae*, genus *Orthohantavirus*, and are enveloped, tri-segmented, single-stranded negative-sense RNA viruses (Jonsson et al., 2010). They cause two distinct, life-threatening clinical syndromes, one being Hemorrhagic Fever with Renal Syndrome (HFRS) in Eurasia, and Hantavirus Cardiopulmonary Syndrome (HCPS) in the Americas (Heyman et al., 2009). The latter carries a severe case fatality rate reaching up to 54.3% (Campos et al., 2009), though higher rates exceeding 70% are occasionally misreported in recent literature (Kumar and John, 2026).

Infection begins when the viral Gn protein docks onto host surface receptors, using integrin and protocadherin-1 (PCDH1) (Gavrilovskaya et al., 1999; Jangra et al., 2018). Once internalized via endocytosis, a drop in endosomal pH forces the viral envelope to fuse with the host membrane, releasing the genome into the cytoplasm. The viral RNA-dependent RNA polymerase (RdRp) starts both transcription and genome replication, before structural proteins and newly synthesized RNA assemble at the Golgi complex or plasma membrane prior to budding out as mature infectious virions (see **Figure 1**) (Hussein et al., 2011; Hepojoki et al., 2012; Vaheri et al., 2013b; Bradfute et al., 2024).

**Figure 1:**
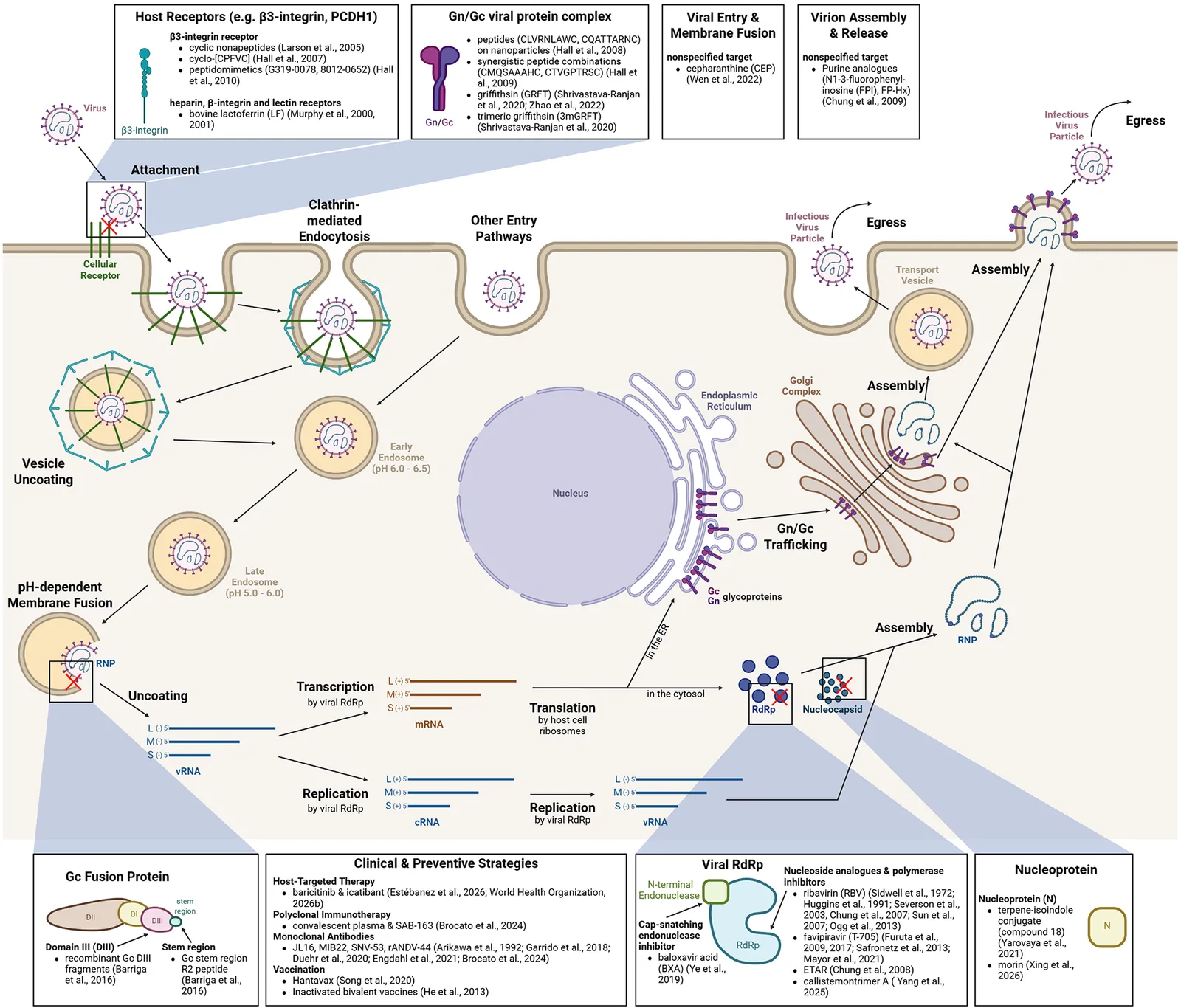
The orthohantavirus life cycle and points of therapeutic intervention. Key stages of cellular infections are shown. Red crosses (X) indicate specific steps targeted by the therapeutic interventions discussed in the review. Adapted from Jonsson et al. (2010); Mir (2010); Vaheri et al. (2013b). cRNA, complementary RNA; L, Large segment; M, Medium segment; mRNA, messenger RNA; PCDH1, Protocadherin-1; RdRp, RNA-dependent RNA polymerase; RNP, Ribonucleoprotein; S, Small segment; vRNA, viral RNA. Created with BioRender.com.

Current orthohantavirus management relies on supportive care and repurposed drugs with limited efficacy. To overcome this public health issue, this review presents existing treatment strategies and advocates for modern *in silico* approaches like molecular docking. Specifically, we highlight the potential of rational drug design, encouraging the systematic virtual screening of diverse chemical and natural product libraries to discover accessible, broad-spectrum hantaviral treatments.

## Viral entry and fusion inhibitors

One of the most promising strategies to inhibit hantaviral entry into host cells is the use of cyclic peptides. With the help of a phage library, where peptide libraries are displayed on the surfaces of filamentous bacteriophages, Larson et al. (2005) identified cyclic nonapeptides that bind the β3-integrin receptor, which pathogenic orthohantaviruses like Sin Nombre virus (SNV), Hantaan virus (HTNV), Seoul virus (SEOV), and Puumala virus (PUUV) bind for viral entry into human endothelial cells. Eleven of the 70 sequences showed inhibitory effects of >80% against SNV *in vitro*, the most potent of these, CPFVKTQLC, demonstrated high inhibitory potency when presented by phage (IC_50_ = 2.7 x 10^8^ particles/µL) (Larson et al., 2005). This peptide was further refined to its minimal functional unit required through alanine substitution and deletion scanning to the cyclic pentapeptide cyclo-[CPFVC] (Hall et al., 2007). However, because free monovalent peptides were less effective than those presented on phage, subsequent research (Hall et al., 2008) shifted towards presenting peptides in a multivalent format on nanoparticles and targeting the orthohantavirus glycoproteins directly rather than the host β3-integrin receptor. The three most potent binders of the virion were selected and attached to 100 nm nanoparticles to create a multivalent inhibitor. When the peptide CLVRNLAWC was presented on a nanoparticle at a 4:1 ratio, its inhibition of SNV jumped from 9% (as a free peptide) to 32.5%. However, the peptide CQATTARNC achieved an even higher efficacy, with inhibition surpassing 50% when the nanoparticle-to-virus ratio was increased to 20:1 (Hall et al., 2008). Building on these strategies, the research was expanded to Andes virus (ANDV). Using a similar approach, Hall et al. (2009) eluted the peptide library from purified ANDV particles using monoclonal antibodies (mAbs) against the Gn and Gc glycoproteins. A major finding in this study was the combined use of peptides that targeted different Gn- and Gc-specific sequences resulted in synergistic inhibition (anti-Gc/Gn peptides CMQSAAAHC and CTVGPTRSC) of infection that was significantly more powerful than single-peptide treatments, emphasizing the benefit of targeting multiple viral epitopes (Hall et al., 2009). Lastly, to search for peptidomimetic molecules to block SNV infection, Hall et al. (2010) used the 3D structure of the previously refined pentapeptide cyclo-[CPFVC], in a ligand-based virtual screening approach to evaluate over 600,000 small molecules. This transition from biological peptides to drug-like peptidomimetics identified compounds such as G319–0078 and 8012-0652, which were 2,000 times more potent than the original cyclic peptides. These molecules demonstrated high-affinity inhibition in the nanomolar range *in vitro* (EC_50_ values between 1.3 - 2.5 nM) (Hall et al., 2010), successfully evolving the initial lead into a highly potent preclinical candidate warranting further *in vivo* evaluation.

Another distinct strategy to prevent infection focuses on disrupting the viral membrane fusion process. Because the orthohantavirus Gc glycoprotein functions as a class II fusion protein, completing membrane fusion requires a major structural relocation of Domain III (DIII) to achieve the post-fusion state (Barriga et al., 2016; Arata-Salas et al., 2026). Exploiting this mechanical vulnerability, Barriga et al. (2016) demonstrated that viral entry can be successfully blocked using recombinant fragments and synthetic peptides derived from the Gc DIII and stem region. These fragments allow initial Gc trimerization to proceed but ultimately inhibit the pathway by preventing membrane hemifusion and fusion pore formation (Barriga et al., 2016). The recombinant DIII fragment inhibited ANDV fusion by around 70% at 25 µM and demonstrated cross-reactivity by reducing PUUV-mediated fusion by 50%. Even greater potency was observed with the R2 peptide, derived from the C-terminal part of the Gc stem region, which inhibited ANDV fusion by up to 95% *in vitro* (Barriga et al., 2016). Although efficacy dropped to a 70% cross-reduction (at 20 µM) against PUUV-mediated fusion (Barriga et al., 2016) due to its high genetic divergence between the two viruses, it still demonstrates significant cross-protective potential.

Another very potent cell-entry inhibitor is Griffithsin (GRFT), an algae-derived lectin that has demonstrated high efficacy against several enveloped viruses such as coronaviruses, retroviruses, lentiviruses, flaviviruses, hepaciviruses, paramyxoviruses, and herpesviruses, with the notable exclusion of gamma- and delta-retroviruses. Interestingly, GRFT is also effective against the non-enveloped human papillomavirus (HPV), where it acts during a late step in the viral entry process rather than during initial attachment (O’Keefe et al., 2010; Lusvarghi and Bewley, 2016). *In vivo* administration of GRFT demonstrated clear therapeutic efficacy, significantly mitigating disease severity and reducing mortality in lethal SARS-CoV infection models (O’Keefe et al., 2010). Highlighting its strong translational potential, GRFT has also advanced to phase I clinical trials against human immunodeficiency virus (HIV-1) infections (ClinicalTrials.gov Identifiers: NCT04032717, NCT02875119). In addition to potent inhibition of ANDV entry (Shrivastava-Ranjan et al., 2020), found that a synthetic trimeric variant (3mGRFT) possessing nine oligosaccharide-binding sites demonstrated even greater *in vitro* potency against ANDV and SNV. However, in contrast to its post-infection efficacy seen in other viral models (Lusvarghi and Bewley, 2016), the authors observed that GRFT lacked potent inhibitory activity against ANDV post-infection. While GRFT showed *in vitro* effectivity against New World hantaviruses (Shrivastava-Ranjan et al., 2020), the lectin inhibited HTNV *in vivo* and provided partial protection in suckling mice infected with HTNV (Zhao et al., 2022). Moreover, GRFT can be produced on a large scale and can be easily purified, highlighting its potential as an antiviral candidate for further preclinical testing against orthohantaviruses (Zhao et al., 2022).

Exploring natural host defense proteins, bovine lactoferrin (LF) has demonstrated significant efficacy as a viral inhibitor against SEOV. LF prevents initial viral adsorption and adhesion, likely by interacting with cellular glycosaminoglycans such as heparin, or by sterically hindering the virus from binding to host β-integrin and lectin receptors (Murphy et al., 2000, 2001). Importantly, LF has no antiviral effect once the virus entered the host cell. *In vivo*, prophylactic administration of LF one day prior to orthohantavirus inoculation improved survival rates in mice up to 70% (at 160 mg/kg), with a double-administration regimen enhancing survival to over 94%. Furthermore, when prophylactic LF treatment was combined with subsequent ribavirin administration, the two agents functioned synergistically, profoundly reducing viral shedding and completely inhibiting viral protein expression (Murphy et al., 2000, 2001).

Discovered through a high-throughput screening of over 2,300 approved drugs and traditional Chinese medicines, cepharanthine (CEP), which is a compound isolated from *Stephania cephalantha* Hayata, has emerged as a highly potent viral entry and membrane fusion inhibitor. *In vitro*, CEP inhibited HTNV pseudovirus (EC_50_ value of 0.9 µM) and reduced viral nucleic acid copies by 73% at 20 µM. *In vivo*, administering CEP to Balb/c mice five hours post-infection significantly reduced infection by 93% (at 90 mg/kg/day) and 94% (at 180 mg/kg/day). Given its established safety profile, CEP represents a high-priority candidate for repurposing and advancement into human clinical trials against orthohantaviruses (Wen et al., 2022).

## Viral replication and polymerase inhibitors

Ribavirin (1-β- D-ribofuranosyl-1H-1,2,4-triazole-3-carboxamide; RBV), a synthetic guanosine nucleoside analogue, serves as the historical benchmark for anti-hantaviral therapy (Sidwell et al., 1972). Once it is transported into host cells, RBV is rapidly phosphorylated into metabolites such as RBV-5’-monophosphate (RBV-MP) and RBV-5’-triphosphate (RBV-TP). While RBV-MP inhibits inosine monophosphate dehydrogenase (IMPDH), resulting in a decrease in intracellular GTP levels, RBV-TP selectively interacts with the viral RdRp as its primary mechanism of action (Severson et al., 2003; Sun et al., 2007). The incorporation of RBV leads to accumulation of genomic errors, and thus to dramatic loss of virus viability (“error catastrophe”) (Severson et al., 2003; Chung et al., 2007). Preclinical evaluation confirmed that early RBV administration reduces viral loads and prevents severe disease in suckling mouse models infected with Hantaan virus (HTNV) and hamster models infected with Andes virus (ANDV) (Huggins et al., 1986; Ogg et al., 2013). Clinically, intravenous RBV demonstrated efficacy in a double-blind, placebo-controlled trial of 242 patients with HTNV-induced HFRS, significantly reducing hemorrhagic and overall mortality (Huggins et al., 1991). However, efficacy varies significantly across strains and clinical syndromes. In the context of HCPS caused by Andes virus (ANDV), intervention with RBV eliminated mortality compared to a 35% historical case-fatality rate and reduced the incidence of the severe cardiopulmonary phase from 84% to 57% (Strella et al., 2025). Conversely, in a larger placebo-controlled, double-blinded trial evaluating intravenous RBV for HCPS, the drug failed to demonstrate overall therapeutic efficacy, revealing no significant difference in survival (70% with RBV vs. 62% with placebo), nor in necessity of extracorporeal membrane oxygenation (ECMO) between groups (Mertz et al., 2004). Trials in HFRS caused by Puumala virus (PUUV) also showed insufficient efficacy and safety of intravenous RBV, likely due to delayed drug administration and weaker correlation between viral load and disease severity compared to HTNV infection (Malinin and Platonov, 2017). Despite its status as an established treatment for HFRS, the clinical utility of RBV is constrained by dose-limiting toxicities, most notably reversible hemolytic anemia, reported in 71% of recipients (Chapman et al., 1999; Jonsson et al., 2010). Furthermore, the lack of efficacy in HCPS and PUUV-caused HFRS is attributed to the rapid clinical progression of these diseases, which often leaves a narrow window for intervention before the onset of irreversible organ failure (Heyman et al., 2009; Vaheri et al., 2013a; Strella et al., 2025).

Originally discovered by Toyama Chemical Co., Ltd., during a screening for anti-influenza compounds, the prodrug favipiravir (T-705) has emerged as a potent viral replication inhibitor. Upon cellular entry, host enzymes convert favipiravir into its active form, favipiravir-RTP. This active metabolite functions as a purine nucleotide analogue that is incorporated into nascent viral RNA, inducing lethal mutagenesis in the viral genome (Furuta et al., 2009, 2017). Safronetz et al. (2013) have shown that favipiravir potently inhibited ANDV and SNV *in vitro*, and provided 100% survival in lethal ANDV hamster models when treatment was initiated before viremia, making it an important drug candidate for post-exposure prophylaxis. To further enhance clinical outcomes, Mayor et al. (2021) combined the use of favipiravir with ribavirin to achieve synergistic orthohantavirus inhibition. First, they determined that favipiravir and ribavirin have similar viral inhibitions against HTNV *in vitro* (favipiravir: IC_50_ value of 3.89 µM, IC_90_ value of 9.79 µM; ribavirin: IC_50_ value of 2.65 µM, IC_90_ value of 10.49 µM). Subsequent combination assays revealed highly synergistic effects, with peak antiviral activity observed at a ratio of 12 µM favipiravir to 18.75 µM ribavirin. This combination successfully reduced HTNV particles, achieving >99% overall inhibition (Mayor et al., 2021). Most recently, Jeyachandran et al. (2025) revealed that favipiravir treatment induces minimal transcriptional changes in host cells. These findings together with established human clinical tolerance, reinforce favipiravir’s status as an important candidate for treating orthohantavirus infections (Jeyachandran et al., 2025).

Broadening the scope of antiviral nucleoside analogues, ETAR (1-β-D-ribofuranosyl-3-ethynyl-[1,2,4]triazole) has emerged as a promising lead compound against both Old and New World hantaviruses. Upon cellular entry, ETAR is metabolized into its active triphosphate form (ETAR-TP). This active metabolite depletes intracellular GTP pools and is thought to either directly inhibit the viral RdRp or induce premature RNA chain termination. ETAR demonstrated high potency *in vitro* against HTNV (EC_50_ value of 10 µM) and ANDV (EC_50_ value of 4.4 µM). Furthermore, *in vivo* studies using HTNV-challenged suckling mice showed that ETAR conferred ~25% protection. Additionally, ETAR achieved this antiviral activity at doses of just 12.5 and 25 mg/kg, which provided similar efficacy to a much higher 50 mg/kg dose of ribavirin. By mirroring ribavirin’s therapeutic outcomes at a fraction of the concentration, these results suggest that ETAR may represent a significantly more effective and optimized antiviral lead compound against orthohantavirus infections (Chung et al., 2008).

Representing a distinct class of purine analogues, N^1^-3-fluorophenyl-inosine (FPI; EC_50_ = 94 µM) and its derivative FP-Hx (EC_50_ = 234 µM) selectively inhibit HTNV. Unlike RBV, FPI does not deplete GTP pools or induce mutagenic error catastrophe, operating instead through post-replication disruption of virion assembly and release (Chung et al., 2009).

Baloxavir marboxil is an FDA-approved influenza therapeutic whose active form, baloxavir acid (BXA), was evaluated against orthohantaviruses *in vitro* by Ye et al. (2019). Because both influenza and orthohantaviruses are negative-sense RNA viruses that rely on host mRNA cap-snatching for viral transcription, they present a similar target for therapeutic intervention. Inside the host cell, the active BXA targets the endonuclease domain of the viral RNA-dependent RNA polymerase (RdRp), inhibiting this crucial-cap snatching activity. Although BXA requires higher concentrations to inhibit hantaviral replication compared to influenza (IC_50_ value of 27.2 µM), it nevertheless efficiently inhibited HTNV replication *in vitro*, establishing it as a promising candidate for future *in vivo* evaluation (Ye et al., 2019).

In the search for novel replication inhibitors, Yang et al. (2025) isolated callistemontrimer A, a phloroglucinol oligomer, from the leaves of *Callistemon rigidus*, a plant with a well-documented history in traditional Chinese medicine. *In vitro* evaluations demonstrated that this compound is exceptionally potent against HTNV (EC_50_ value of 0.65 µM), outperforming the standard reference drug RBV (EC_50_ value of 13.16 µM) by a factor of ~20 under identical conditions. Molecular docking analyses indicate that callistemontrimer A binds directly to the active site of the viral RdRp. By forming five stable hydrogen bonds within its active site, the compound effectively blocks viral genome synthesis (Yang et al., 2025).

Beyond targeting the polymerase, the viral nucleoprotein (N) has emerged as a promising alternative target for small-molecule intervention (Yarovaya et al., 2021). identified a new class of inhibitors by conjugating natural terpene scaffolds, such as camphor or fenchone, with an artificial isoindole fragment. The lead candidate, compound 18, demonstrated potent anti-HTNV activity (IC_50_ = 7.6 µM) and exceptionally low toxicity (CC_50_ > 1,000 µM) *in vitro*. Pseudovirus studies and molecular docking suggest they bind to the highly conserved RNA-binding site of the nucleoprotein, and by competing with viral RNA for this site, these inhibitors effectively arrest intracellular replication, offering a specialized approach to disrupt the viral assembly machinery (Yarovaya et al., 2021).

Another compound that disrupts nucleocapsid assembly is morin, a natural flavonoid found in plants like mulberry and perilla. Known for its diverse biological and anti-influenza properties, morin effectively inhibits HTNV *in vitro* (IC_50_ value of 8.03 µM). The compound binds directly to the core assembly region of the viral nucleoprotein. This sterically hinders RNA encapsidation and overall nucleocapsid assembly, disrupting the formation of functional replication complexes during the post-entry stage of infection (Xing et al., 2026).

## Beyond antivirals: supportive care, immunotherapy, and vaccination

In the absence of licensed antiviral treatments, clinical management remains primarily supportive, focusing on intensive monitoring and treating of respiratory, cardiac, and renal complications to improve outcomes (World Health Organization, 2026a). Achieving an early diagnosis remains a primary clinical challenge, as the efficacy of supportive care is substantially reduced if initiated after the onset of severe disease. This highlights the critical need to increase clinical awareness, strengthen early diagnostic capacity, and recognize suspected cases promptly, particularly in endemic areas of orthohantavirus circulation. Host-targeted and anti-inflammatory strategies have recently been explored to mitigate the characteristic vascular leak and cytokine storm. Most notably, during the 2026 cruise ship outbreak, the JAK inhibitor baricitinib and the bradykinin B2 receptor antagonist icatibant were administered in a combined therapeutic regimen alongside RBV and favipiravir (Estébanez et al., 2026; World Health Organization, 2026b). Passive immunotherapy also continues to be a major investigative focus. While convalescent plasma has shown potential to reduce mortality, newer candidates, such as the quadrivalent human polyclonal antibody SAB-163 and highly potent monoclonal antibodies (e.g., JL16, MIB22, SNV-53, and rANDV-44), have demonstrated up to 100% protection in lethal animal models (Arikawa et al., 1992; Garrido et al., 2018; Duehr et al., 2020; Engdahl et al., 2021; Brocato et al., 2024). Regarding prevention, Hantavax has been used to prevent HFRS in South Korea in previous decades (Song et al., 2020), and China routinely administers inactivated bivalent vaccines targeting HTNV and SEOV in endemic regions (He et al., 2013). However, a globally licensed orthohantavirus vaccine does not currently exist, leaving New World strains and HCPS without an approved preventative countermeasure.

## Conclusion

The persistent threat posed by orthohantaviruses underscores the absence of targeted, licensed antivirals. While clinical management currently relies on supportive care and repurposed drugs, long-term solutions require rational drug design aimed at key steps in the viral life cycle, especially Gn-mediated viral entry and RdRp-driven genome replication. Modern *in silico* approaches, particularly molecular docking and virtual screening, offer powerful tools to bridge this therapeutic gap by screening extensive chemical libraries. Broad chemical libraries, including natural product derivatives, represent an exceptionally rich, structurally diverse reservoir for drug discovery. However, while most current research heavily targets well-studied strains like HTNV, SEOV, and ANDV, numerous other pathogenic orthohantaviruses remain largely neglected. Expanding future *in vitro* and *in vivo* testing to evaluate candidate compounds against these neglected strains will be essential to develop broad-spectrum hantaviral therapeutics capable of mitigating future epidemic threats.
